# Improved Resolution of Reef-Coral Endosymbiont (*Symbiodinium*) Species Diversity, Ecology, and Evolution through *psbA* Non-Coding Region Genotyping

**DOI:** 10.1371/journal.pone.0029013

**Published:** 2011-12-28

**Authors:** Todd C. LaJeunesse, Daniel J. Thornhill

**Affiliations:** 1 Department of Biology, Pennsylvania State University, Mueller Laboratory, University Park, Pennsylvania, United States of America; 2 Defenders of Wildlife, Washington, District of Columbia, United States of America; Université Paris Sud, France

## Abstract

Ribosomal DNA sequence data abounds from numerous studies on the dinoflagellate endosymbionts of corals, and yet the multi-copy nature and intragenomic variability of rRNA genes and spacers confound interpretations of symbiont diversity and ecology. Making consistent sense of extensive sequence variation in a meaningful ecological and evolutionary context would benefit from the application of additional genetic markers. Sequences of the non-coding region of the plastid psbA minicircle (*psbA^ncr^*) were used to independently examine symbiont genotypic and species diversity found within and between colonies of Hawaiian reef corals in the genus *Montipora*. A single *psbA^ncr^* haplotype was recovered in most samples through direct sequencing (∼80–90%) and members of the same internal transcribed spacer region 2 (*ITS2*) type were phylogenetically differentiated from other *ITS2* types by substantial *psbA^ncr^* sequence divergence. The repeated sequencing of bacterially-cloned fragments of *psbA^ncr^* from samples and clonal cultures often recovered a single numerically common haplotype accompanied by rare, highly-similar, sequence variants. When sequence artifacts of cloning and intragenomic variation are factored out, these data indicate that most colonies harbored one dominant *Symbiodinium* genotype. The cloning and sequencing of *ITS2* DNA amplified from these same samples recovered numerically abundant variants (that are diagnostic of distinct *Symbiodinium* lineages), but also generated a large amount of sequences comprising PCR/cloning artifacts combined with ancestral and/or rare variants that, if incorporated into phylogenetic reconstructions, confound how small sequence differences are interpreted. Finally, *psbA^ncr^* sequence data from a broad sampling of *Symbiodinium* diversity obtained from various corals throughout the Indo-Pacific were concordant with *ITS* lineage membership (defined by denaturing gradient gel electrophoresis screening), yet exhibited substantially greater sequence divergence and revealed strong phylogeographic structure corresponding to major biogeographic provinces. The detailed genetic resolution provided by *psbA^ncr^* data brings further clarity to the ecology, evolution, and systematics of symbiotic dinoflagellates.

## Introduction

The acquisition of genetic evidence continues to further our understanding of the ecology and evolution of dinoflagellates in symbioses with reef-building corals and many other invertebrates [1]–[4]. Sequences of ribosomal and single-copy nuclear, chloroplast, and mitochondrial DNA indicate that the ancient symbiont genus *Symbiodinium* comprises highly divergent groups (i.e., Clades) each containing a diversity of genetically distinct lineages (reviewed in [4]). The habitat and biogeographic distributions of particular host-symbiont combinations allude to underlying ecological and evolutionary processes shaping the fidelity and flexibility of these associations [5], [6]. However, many *Symbiodinium* that exhibit ecological differences in host specificity are closely related and differentiated by only small, albeit fixed, numbers of nucleotide changes in conventional nuclear and plastid DNA sequences.

Despite the availability of informative genetic markers, there is presently little consensus on how to classify *Symbiodinium* species diversity without relying on traditional morphological traits (cf. [7]). While internal transcribed spacer region 2 (*ITS2*) data are commonly used to define the diversity of these symbionts [6]–[12], the finding of extensive intra-specific sequence variation among *ITS* regions reported in free-living dinoflagellates [13] challenges assertions that even a fixed single-base substitution or insertion-deletion (indel) is sufficient to characterize a species of *Symbiodinium* [7]–[9], [11], [12]. Despite this, it was recently demonstrated that a lineage-based approach [14], [15] combining sequences of mitochondrial (*Cyt b*), chloroplast (*cp23S*) and ribosomal genes (*LSU*) and spacer regions (*ITS*) could identify and classify *Symbiodinium* into fundamental biological units (i.e., species) [7]. Whereas the sequence analysis of a single gene barely resolves distinct lineages, additional sequence information improves phylogenetic resolution by increasing branch length separation and branch support among actual species lineages. Alternatively, sequences from microsatellite flanker regions and the non-coding regions of the dinoflagellate plastid minicircles, may have greater resolving power that unambiguously delimit closely-related taxa without relying on the assembly of numerous independent sequences.

The chloroplast genes of peridinin-containing dinoflagellates occur on separate plasmid-like minicircles of 2–5 kbp. Each contains a non-coding region that may function in gene replication and transcription [16]–[19]. Preliminary analysis of *psbA* minicircles among *Symbiodinium* observed extensive variation in the non-coding regions (*psbA^ncr^*) among isolates of Clade C [20] and were non-alignable with sequences from other Clades (e.g., A and B). Moreover isolates with similar or identical large sub-unit (*LSU*) rDNA sequences also shared similar *psbA^ncr^* sequences suggesting a concordance with nuclear gene evolution [20]. Indeed, this plastid maker shows great promise for investigations of *Symbiodinium* ecology, evolution, and systematics. In a recent application, *psbA^ncr^* sequence phylogenies unequivocally resolved three host-specific Clade C *Symbiodinium* lineages corresponding to each of three *Pocillopora* spp. in the eastern tropical Pacific [21]. These lineages were identified as closely-related, yet separate, *ITS2* types using denaturing gradient gel electrophoresis (DGGE) [8]. Therefore, the *psbA^ncr^* may definitively resolve independently evolving *Symbiodinium* spp. that are initially diagnosed, but are not well resolved, by *ITS* genotyping.

In order to more thoroughly assess the utility of the *psbA^ncr^*, we examined this marker in the context of *Symbiodinium* diversity among common Hawaiian reef corals in the genus *Montipora*. Symbiont diversity in *M. capitata* from the island of O'ahu was examined in two previous studies with conflicting results. Based on PCR-DGGE analysis of the *ITS2* region, LaJeunesse et al. [22] reported symbioses between Hawaiian *M. capitata* and two *Symbiodinium ITS2* lineages: type *D1a* (in orange colony morphs at depths of 1–2 m; now more precisely referred to as *D1-4-6*; *sensu* [12]) and type *C31* (involving various colony morphologies distributed across a depth range of 1–5 m). In contrast, a recent study employing bacterial cloning of the *Symbiodinium ITS2* in *M. capitata* from shallow (1–2 m) habitats in O'ahu reported 17 different *ITS2* variants, including sequences representing *D1/D1a*, *C31*, *C21*, *C3*, *C17* and 11 additional, closely-related sequences at lower frequency [23]. There are thus two competing hypotheses for symbiotic associations in *M. capitata* based on the application of two different methodologies (i.e., PCR-DGGE vs. PCR and bacterial cloning) analyzing *ITS2*. The first paradigm indicates that coral colonies are often dominated by a single symbiont *ITS2* type with symbiont species diversity partitioned primarily among different host species, habitats, and/or depths [22]. The alternative paradigm suggests that communities comprising many different symbiont *ITS2* types occur within each colony with overall endosymbiont diversity greater within than between host individuals [23].

Resolution of these alternate possibilities is paramount to understanding the ecology and evolution of coral-algal symbioses. The opposing interpretations of *Symbiodinium* diversity between LaJeunesse et al. [22] and Stat et al. [23] may result from the multi-copy and heterogeneous nature of eukaryotic ribosomal genes and spacer regions and the different methodologies used to analyze this maker for ecological studies [7], [24]. When applied to a multi-copy gene array, the more-conservative approach of PCR-DGGE screening targets the dominant copies of the ribosomal array [24], [25], whereas cloning generates large amounts of sequence data that include mixtures of both dominant and rare, functional and non-functional, intra-genomic and inter-genomic sequence variants combined with PCR and cloning artifacts [7], [24]. By comparison, plastid DNA markers, such as the *psbA^ncr^*, are more akin to single-copy loci in that they exhibit relatively low intragenomic variation and their use may reduce uncertainty when evaluating genetic diversity. We therefore used the *psbA^ncr^* to test the competing hypotheses of LaJeunesse et al. [22] and Stat et al. [23]. The *psbA^ncr^* and *ITS2* were PCR-amplified, bacterially cloned, and sequenced in a subset of samples. Cloned *ITS2* sequences were compared with results from parallel analyses using DGGE screening and direct sequencing of numerically abundant *ITS2* variants observed as bright bands in the gel profiles (i.e., fingerprints) of symbionts associated with three *Montipora* spp. from Hawaii. Furthermore, the *psbA^ncr^* was cloned and sequenced from several isoclonal cultures of Clade C to assess intragenomic variation. Single copies of cloned *psbA^ncr^* were then re-amplified, re-cloned, and sequenced to determine the relative contribution of mutation artifacts generated by the PCR and cloning process. Finally *psbA^ncr^* sequences from the *Symbiodinium* in *Montipora* and other common reef coral genera at selected locations throughout the Indo-Pacific were evaluated phylogenetically for concordance with DGGE-*ITS2* genotyping.

## Materials and Methods

### Sample collection, processing and preservation

Coral tissue samples used in this investigation were collected using scuba equipment from deep and shallow reef habitats from O'ahu, Hawaii [24], the Great Barrier Reef, Australia [26], [27], Zamami Island, Japan [27], Zanzibar, Tanzania [12], and the Andaman Sea, Thailand [12]. *Montipora* spp. samples examined in detail from O'ahu, Hawaii were collected in July of 2002 from the shallow lagoon (1–5 m) and deep fore-reef environments (20 m) of Kanehoe Bay, and from intermediate depths from a site on the north shore of O'ahu. DNA samples used in this study were from previously published archived samples. Hawaiian samples were acquired under collecting permits issued from the State of Hawaii to the Hawaii Institute of Marine Biology during the Edwin W. Pauley summer course “Molecular Biology of Corals” in 2002. The Great Barrier Reef Marine Park Authority provided permits to collect under the auspices of the Australian Institute of Marine Science and University of Queensland. Collections from the Andaman Sea were permitted by the National Research Council of Thailand (no. 0002.3/5154).

Samples were processed by stripping animal tissue from their skeletons using high-pressure bursts of air and then separating the dense symbiont cells using centrifugation [see 26 for details]. The resulting pellet was preserved in a sodium salt-DMSO buffer [28]. Alternatively, whole skeletal fragments with live tissue were preserved. A supplementary table is provided that details the coral species sampled, collection dates, locations, specific sites surveyed at each location, colony depth, the host genus and species, as well as GenBank accession numbers for each *psbA^ncr^* haplotype sequence (Table S1).

Three iso-clonal *Symbiodinium* cultures, rt113, rt152, and rt203 (“rt” is used here to indicate original cultures from the Robert K. Trench collection, [2]), were chosen to assess the degree of intragenomic varation for *psbA^ncr^*. All are representatives of Clade C; culture rt113 is the holotype of *Symbiodinium goreaui* (Trench and Blank 1987) isolated from the corallimorpharian *Rhodactis lucida* in the Caribbean; culture rt152 shares the same *ITS2* type (C1) with *S. goreaui* and was isolated from the corallimorph *Discosoma sanctithomae*; whereas culture rt203 was isolated from the giant clam *Hippopus hippopus* from Palau and represents a different *ITS2* lineages (type C2 *sensu* [2]). *Symbiodinium* cells from each of these cultures were concentrated by centrifugation and preserved in sodium salt-DMSO buffer [28].

### Molecular-genetic analysis

Preserved *Symbiodinium* pellets or skeletal fragments comprising approximately 5–10 mm^2^ of preserved tissue were mechanically disrupted using a Biospec Beadbeater (Bartlesville, OK, USA) for 1 min at maximum speed. Nucleic acids were then extracted using a modified Wizard DNA extraction protocol developed by Promega (Madison, WI, USA; see LaJeunesse et al. [12], [26] for protocol modifications). All samples were initially analyzed using denaturing gradient gel electrophoresis fingerprinting (i.e., genotyping) and sequencing of the ribosomal internal transcribed spacer 2 (*ITS2*) as described by LaJeunesse [8]. This technique is employed to target the numerically dominant sequence variant(s) in a sample [24]. The host mitochondrial control region was amplified and directly sequenced for two suspect samples using the ms_FP2 and MON_RP2 primers and PCR conditions described by van Oppen et al. [29]

Samples were further analyzed by amplifying and directly sequencing the non-coding region of the plastid *psbA* minicircle using the primers (7.4-Forw, 5′ - GCA TGA AAG AAA TGC ACA CAA CTT CCC - 3′, and 7.8-Rev, 5′ - GGT TCT CTT ATT CCA TCA ATA TCT ACT G - 3′ [17]). These primers preferentially amplify Clade C *psbA* ^ncr^ haplotypes over those from Clade D. As an alternative, the universal primers **psbAFor_1** (5′- GCA GCT CAT GGT TAT TTT GGT AGA C - 3′) and **psbARev_1** (5′- AAT TCC CAT TCT CTA CCC ATC C - 3′) were used to amplify *psbA* ^ncr^ haplotypes of clade D. The PCR conditions for all amplifications were: 94°C for 2 min; then 40 cycles of 94°C 10 s, 55°C for 30 s and 72°C for 2 min; followed by a final extension at 72°C for 10 min. Samples appearing homogenous for *D1-4-6* based on *ITS2*-DGGE profiles from *M. capitata* and *M. patula* were used [22]. Finally, amplifications of *ITS2* and *psbA^ncr^* sequences from genomic extracts of five *Montipora capitata* samples collected in Hawaii were cloned using a pGEM-T cloning kit (Promega) following the manufacturer's instructions. For each genetic marker 9–15 clones were sequenced.

PCR products were usually sequenced in one direction using Big Dye 3.1 reagents (Applied Biosystems, USA) at the DNA Core Facility (Penn State University) using the reverse primer from the *ITS2* (ITSrev) and forward primer for the psbA (7.4-Forw). Chromatograms were visually inspected for accuracy in base calling (Sequence Navigator) and the edited sequences aligned initially using the online application of ClustalW2 (http://www.ebi.ac.uk/Tools/msa/clustalw2/) and additional adjustments to the alignment made during visual inspection of the output file using MacClade version 4.06 (Sinauer & Associates).

To examine the amount of *psbA^ncr^* variation within a *Symbiodinium* genome (i.e., intragenomic variation), the *psbA^ncr^* was amplified from isoclonal cultures rt113, rt152, and rt203 using the 7.4-Forw and 7.8-Rev primers, the product cloned, and then bi-directionally sequenced (see above). A total of 16 clones were originally sequenced from each culture. Based on these data a second round of cloning was conducted where one representative clone from each culture was selected, re-cloned, and then sequenced repeatedly (n = 16) to determine what proportion of sequence variation can be attributed to artifacts generated by PCR and/or the process of cloning.

### Data analyses

Phylogenetic reconstructions based on *ITS2* and partial sequences of the *psbA* were conducted using Maximum parsimony (MP) and Distance under the default settings in PAUP* 4.0b10 [30]. For calculating phylogenies based on MP, insertions and deletions (indels) were analyzed as a 5^th^ character state. In analysis of Distance the SYM+I+G model for molecular evolution, a symmetrical nucleotide substitution model incorporating a gamma distribution of rate variation and a proportion of invariant sites was chosen based on Akaike Information Criterion (AIC) in MrModelTest version 2 [31]. To assess statistical significance of internal branching, 1,000 Bootstrap replicates were performed as well as Bayesian posterior probabilities calculated using MrBayes version 3.12 [32]. Two million generations were analyzed under the SYM+I+G model of sequence evolution, beginning with an unspecified tree topology and no defined prior probabilities. Two sets of four chains (three hot, one cold) were run for 2×10^7^ generations and sampled every 100 generations. In all cases, the chains converged within 2.5×10^6^ generations. Therefore, the first 2,500 trees (2.5×10^6^ generations) were discarded as burn-in and a 50% majority-rule consensus tree was calculated from the remaining 17,500 trees. Nodal support was reported as posterior probabilities.

## Results and Discussion

### *ITS* sequence variation in recognizing *Symbiodinium* diversity

Ribosomal spacer regions are frequently targeted in molecular systematics to delimit species of animals, plants, fungi, macro-algae, and eukaryotic microbes [33], [34]. The current understanding of *Symbiodinium* species diversity, ecology, and evolution is based primarily on analysis of *ITS2* and to some extent *ITS1* sequence data acquired from a broad range of host species from tropical and subtropical regions throughout the world (e.g. [6], [10], [35]–[37]). As with almost all eukaryotes, the multi-copy ribosomal arrays of *Symbiodinium* genomes evolve in concert and are usually dominated by one or two numerically common and very similar sequence variants [24], [38], [39]. For this reason, denaturing gradient gel electrophoresis of the *ITS2* is often used to isolate and identify (1) the dominant intragenomic *ITS2* sequence(s) diagnostic of a particular symbiont lineage (e.g. [7], [8], [24]) and (2) to detect the presence of two or more symbionts co-occurring within a sample (e.g., [8], [40], [41]).

**Figure 1:**
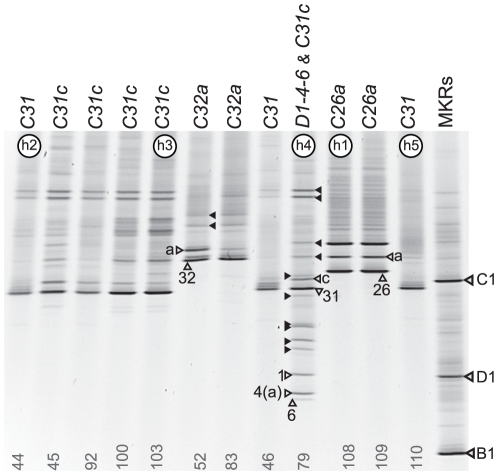
*ITS2*-DGGE fingerprints of *Symbiodinium* from *Montipora* spp. sampled in Oahu, Hawaii. Each diagnostic fingerprint comprises high-melting-lower-migrating homoduplexes (open arrowheads) and low-melting-higher-migrating heteroduplexes (solid arrowheads). Homoduplexes apparently correspond to the numerically dominant sequence variant in the ribosomal array of each ITS type [i.e.], [ 7,24]. Sample 79 contains two symbiont profiles, *C31* and *D1-4-6*. Samples used in bacterial cloning/sequencing of the *ITS2* and *psbA* ^ncr^ are labeled on the gel by their *psbA^ncr^* haplotypes (h1, h2, h3 etc…; see Figs. 2a and 3).

*Montipora* spp. samples from O'ahu, Hawaii were screened using *ITS2*-DGGE to examine the genetic diversity of *Symbiodinium* (Fig. 1). Four distinctive symbiont types were identified, including *C32a* from a shallow (4 m) and deep (10 m) colony of *M. flabellata*, *C26a* from deep (>20 m) encrusting colonies of *M. incrassata* (which were originally misidentified as *M. capitata* in LaJeunesse et al. [22], but were re-identified by mitochondrial control region sequences [29]; JQ043552), and *C31* and *D1-4-6* (originally *D1a* and later changed to *D1a-f* by Smith et al. [42]) from shallow (1–4 m) colonies of *M. capitata*. Many of the *C31* profiles contained a second band designated as “c” (Fig. 1) and may represent a co-dominant intragenomic *ITS2* variant (see [24]), or possibly the presence of a second close-related symbiont. A mixed symbiont population comprising *C31c* and *D1-4-6* was detected in only one sample (sample 79, lane 9 of Fig. 1).

The approach of using DGGE to screen the *ITS2* sequence variation assumes that (1) most samples contain a single dominant symbiont genotype and (2) that the resulting DGGE fingerprint, sometimes comprising multiple bright bands in a profile, generates a visualization of the evolutionary state (i.e., the degree of homogenization through concerted evolution) of the resident symbiont's ribosomal array. If these underlying assumptions were generally wrong, interpretations of DGGE profiles would under assess the diversity of *Symbiodinium in hospite*. Indeed, DGGE screening does not detect background symbiont populations comprising less than 10–15% of the total population [40], [43], [44], including populations that are of potential ecological importance [45]. Extensive *ITS* sequence diversity was reported for *Symbiodinium* in a recent analysis of *M. capitata* colonies from Kaneohe Bay, Hawaii, using bacterial cloning and sequencing [23]. Stat et al. acknowledged that the significance of this sequence diversity was open to several interpretations; however, the authors favored the idea that it represented a combination of several coexisting symbiont lineages within a colony and intragenomic variation from those various symbiont types [23]. To further explore this possibility, we reexamined five of the *Montipora* spp. samples analyzed by DGGE (above) via cloning and extensive sequencing of *ITS2* amplicons. The selected samples included *M. captiata* colonies identified with *C31* (n = 2), *C31c* (n = 1), a mixed community of *C31c*/*D1-4-6* (n = 1), and one *M. incrassata* colony identified with *C26a* (selected samples are designated as h1–h5 in Fig. 1).

**Figure 2:**
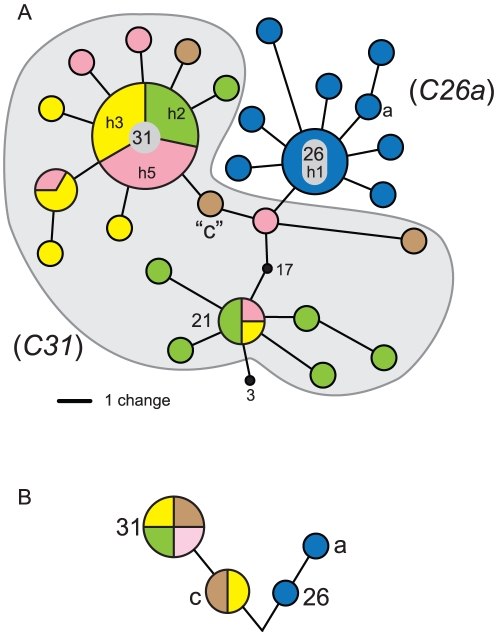
Sequence variation recovered by cloning vs. DGGE screening. (a) Unrooted maximum parsimony of sequence variants recovered from the process of bacterial cloning/sequencing of *ITS2* rDNA from four samples of *C31/C31c* (h2–h5) and one sample of type *C26a* (h1) from a subset of samples presented in Figure 1 (b) A corresponding and highly simplified phylogeny based on screening the numerically dominant sequence variants using DGGE fingerprinting. Clones from each sample were color coordinated for comparison and apply to other figures throughout the text.

**Figure 3:**
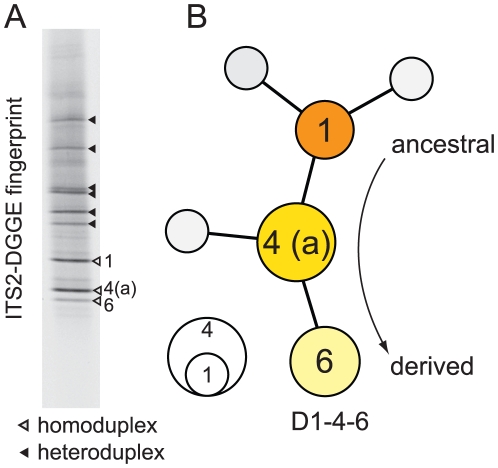
The clade D *Symbiodinium* sp. harbored by Hawaiian *Montipora* (a) *ITS2*-DGGE fingerprinting produced three diagnostic homoduplex bands from each sample containing this *Symbiodinium*. Designated *D1-4-6*, band “1” is ancestral while bands “4” and “6” represent derived sequence variants abundant in the genome. (b) All three dominant sequences were recovered multiple times from the bacterial cloning/sequencing from the sample 79, Figure 1. A profile matching *Symbiodinium D1-4-6* was found in a few *Pocillopora* from the Andaman Sea, northeastern Indian Ocean and was also recovered from *Pocillopora* colonies in the aquarium trade.

Of the 70 cloned *ITS2* fragments sequenced from 5 coral colonies, a total of 33 distinct variants were identified (Figs. 2, 3; GenBank accession numbers JQ003824-JQ003850). This included 27 sequence variants from Clade C and 6 sequence variants from Clade D. Maximum Parsimony analyses showed that within each *Montipora* sample, genetically distinct sequences (color coordinated to identify the sample of origin in Figs. 2, 3) differed by 1–8 base substitutions. All cloned sequences of *C26a* from *M. incrassata* (blue sequences, Fig. 2) clustered together with no phylogenetic overlap with sequences derived from the *M. capitata C31*/*C31c* samples (yellow, green, pink, and brown sequences, Fig. 2). The most commonly recovered sequence from each sample usually occupied a central “ancestral” position from which mostly rare singletons radiated. Furthermore, the most commonly cloned sequences typically corresponded with sequences of the bands from the diagnostic DGGE profiles of each sample (Fig. 1). One exception was the finding of the sequence diagnostic of *Symbiodinium* type *C21* in three of the four samples (n = 4 out of 55 clones), whereas previously only clade C types, *C31* or *C31c*, were detected by DGGE analysis (Fig. 1). Most (12 of 15; 80%) of the cloned sequences recovered from sample 79 were from Clade D and analysis of them is discussed below.

**Figure 4:**
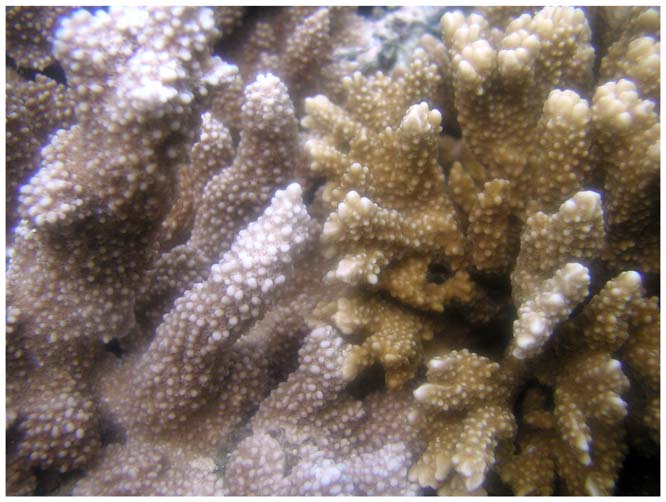
Color morphs of *Montipora capitata* are dominated by different species of *Symbiodinium*. The reddish brown colony on the left harbors the *Montipora*-specific symbiont *C31/C31c*, while the orangish-brown colony on the right harbors the Clade D symbiont, *D1-4-6*.

*Montipora capitata* in Hawaii occasionally harbor Clade D *Symbiodinium* [5], [22]. Preliminarily designated *D1a* by LaJeunesse et al. [22], further examination of the DGGE profile identified a third co-dominant sequence as diagnostic of this *Symbiodinium* type from other members of Clade D (Fig. 3a; designated *D1a-f* by Smith et al. [42]; and renamed *D1-4-6* by LaJeunesse et al. [12]. Colonies of *M. capitata* dominated by *D1-4-6* have an orange hue while those associated primarily with *C31* or *C31c* are red-brownish in coloration (Fig. 4). Sample 79 harbored a mixture of both symbionts (Fig. 1) and when the *ITS2* amplification from this sample was cloned, the majority of sequences were representative of *D1-4-6* (Fig. 3b; JQ003816-JQ003823). Specifically, the three most commonly cloned sequences corresponded to the three bands diagnostic of this symbiont's DGGE profile (i.e., D- “1”, “4” and “6”), with three additional sequences detected as singletons. Stat et al. [23] also detected the same three diagnostic sequences of *D1-4-6*, but interpreted these sequences as potentially representative of three distinct symbionts.

The *ITS2* sequence diversity derived from bacterial cloning (n = 33) was considerably greater than results from DGGE fingerprinting/screening (n = 7, representing only 4 entities; see also [24]). This incongruence between methods mirrors the findings of Stat et al. [23] compared to LaJeunesse et al. [22]. Furthermore, this discrepancy raises fundamental questions about the biology of coral-algal symbioses and the accuracy of the existing genetic taxonomy of *Symbiodinium* (*sensu* [8]). Are the additional sequences recovered by bacterial cloning important to understanding the ecology of these symbioses? Or, do they mostly comprise intra-genomic variants as well as PCR and cloning artifacts, as indicated by Thornhill et al. [24] and Sampayo et al. [7]? Alternatively, is the cloned sequence diversity indicative of diverse *Symbiodinium* species populations found within individual coral colonies (*sensu* [46])? Resolving these questions is critical for the proper interpretation of endosymbiont sequence diversity and for understanding the basic biological processes underpinning the ecology and evolution of coral-algal symbioses. The rapidly evolving non-coding region of the plastid *psbA* minicircle (*psbA^ncr^*) is a candidate marker with low intragenomic variation that may provide independent data to examine symbiont diversity and assess the degree to which colonies host multiple genotypes.

### Resolving *Symbiodinium* diversity with a chloroplast minicircle non-coding region

The plastid genomes of dinoflagellates are highly unusual in that individual genes typically occur independently on small (2–5 kb) plasmid-like minicircles [19]. The total number of genes retained in the chloroplast is small and includes those encoding proteins important in the reaction center of photosystem II, photosystem I, the b6f complex, ATP synthase, as well as rRNA genes [47], [48]. Each minicircle contains a non-coding region of differing length and nucleotide composition that is speculated to function in gene duplication and/or transcription [49]. Recently, sequence comparison of *psbA* minicircle non-coding region (*psbA* ^ncr^) observed substantial divergence among samples of Clade C *Symbiodinium* originating from different hosts [17], [20], [21]. Sequences of the non-coding region are not comparable between members of different Clades and therefore, the use of this genetic marker must be restricted to intra-cladal comparisons [20].

**Figure 5:**
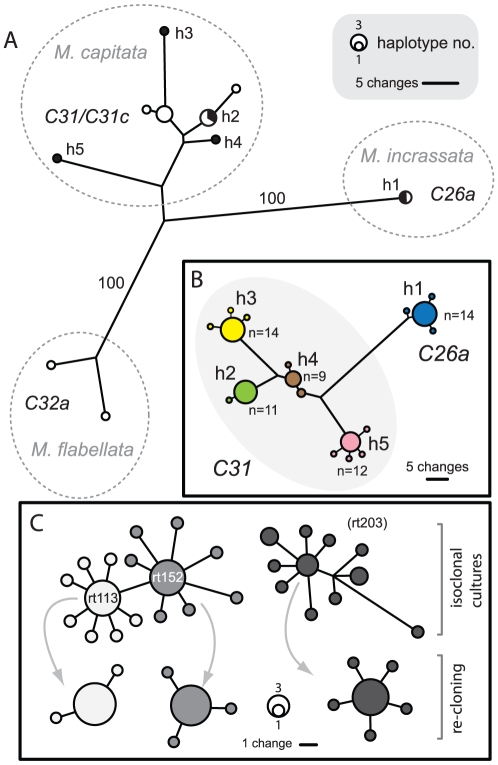
The analysis of *Symbiodinium psbA^ncr^* acquired from field samples and isoclonal cultures. (a) Unrooted phylogenetic analysis of partial (∼500 bases) *psbA* ^ncr^ sequences recovered from direct sequencing of *Symbiodinium* from Hawaiian *Montipora*. The symbiont's ITS2 designation and host species identity are labeled and correspond with phylogenetic groupings based on *psbA^ncr^* sequences. Samples subjected to bacterial cloning and intensive sequencing are shaded in black. Bootstrap values based on 1000 replicates are labeled for branches separating each lineage group. (b) The phylogenetic analysis of bacterially cloned *psbA^ncr^* shows minimal sequence variation within a particular sample, but considerable variation between samples. The number of clones sequenced is indicated for each sample analyzed. (c) The extent of intragenomic variation was examined by the cloning and sequencing of *psbA* ^ncr^ amplicons from the isoclonal clade C cultures, rt113, rt152, and rt203 (n = 16 sequences per culture). Sequence divergence among variants was small among strains of *Symbiodinium goreaui* (type *C1*; rt113 and rt152), however these were nonalignable with sequence variants recovered from rt203. The re-cloning and sequencing (n = 16) of a single cloned amplicon from each cultured isolate (grey arrows) assessed the frequency of artifacts created by the PCR/cloning process.

Direct sequencing of *psbA* ^ncr^ from 16 samples of Hawaiian *Montipora* spp. produced a single unambiguous sequence per sample. No secondary peaks were detected in the chromatograms, suggesting that (1) the *psbA* ^ncr^ exhibits low intragenomic variability (i.e., equivalent to a single-copy marker) in these *Symbiodinium* and (2) each sample contained a single *Symbiodinium* haplotype. The phylogenetic analysis of partial *psbA* ^ncr^ sequences (ranging between 536 and 601 bp in length) produced three divergent groupings. Haplotypes from each phylogenetic grouping shared the same *ITS*-DGGE fingerprint (i.e., *ITS2* type C31/C31c, C26a, or C32a) indicating a correspondence between nuclear and plastid makers (Fig. 5a). Finally, each symbiont lineage associated with a particular species of *Montipora* indicating marked differences in their host specificity and ecological distribution (Fig. 5a).

Because direct sequencing may not detect the presence of background co-occurring *psbA^ncr^* haplotypes, amplifications were cloned and sequenced from the same five samples whose *ITS2* diversity was analyzed using this same approach (see above). Totals of 9 to 14 clones were sequenced from each sample and of these, two to five distinct sequences were recovered. The combined analyses of all these data produced five distinct, non-overlapping sequence clusters (Fig. 5b; JQ043553- JQ043585). The numerically dominant *psbA* ^ncr^ sequence detected in each sample was identical to the sequence obtained by direct sequencing. The remaining cloned sequence variants were singletons that differed from the dominant haplotype by 1 or 2 nucleotide substitutions. In comparison to the cloning of *ITS2* rDNA, the *psbA^ncr^* exhibits less within-sample heterogeneity (i.e., 2–5 sequences were detected per sample for the *psbA* ^ncr^ vs. 6–9 sequences detected per sample for the *ITS2*), while providing unambiguous phylogenetic resolution. The minimal sequence divergence of rare *psbA* ^ncr^ variants suggests that some were created by mutations introduced through polymerase error during PCR or the cloning process (i.e., methodological artifact/error [24]), but alternatively, they could represent low levels of intragenomic variation and/or the existence of closely-related background haplotypes.

The copy number of each minicircle gene is unknown and may vary depending on the growth phase of a dinoflagellate cell [50]. As a result, intragenomic *psbA^ncr^* variation may exist and confound interpretations about diversity (*sensu* [24]). To address this possibility, we PCR amplified, cloned, and sequenced the *psbA^ncr^* from three iso-clonal cultures of Clade C *Symbiodinium* including the holotype of *S. goureai* (rt113 designated *ITS2* type *C1*) and type *C2* (*sensu* [2]), an unnamed species. Results from these analyses show that, when PCR and cloning artifacts are factored out (see below), intragenomic variation does exist in the *psbA^ncr^* and that the extent of this variation may differ between clonal lineages and species of *Symbiodinium* (Fig. 5c; JQ043677- JQ043719). The dominant *psbA^ncr^* sequence of culture rt152 (*ITS2* type *C1*) differed from culture rt113 (*ITS2* type C1) by one base substitution and four insertion-deletions over the entire non-coding region (922 aligned bases). Culture rt152 therefore represents a distinct haplotype variant of *S. goreaui*. The *psbA^ncr^* sequences obtained for rt203 (*ITS2* type *C2*) were not alignable with the haplotypes of *S. goreaui* indicating that this marker has limited phylogenetic utility when comparing *Symbiodinium* spp. whose *ITS-5.8S* regions differ by approximately 2% or more (Fig. S1). Although variation was minimal in the genomes of each *S. goreaui* haplotype, the culture of type *C2* (rt203) contained numerous variants with large indels (Fig. 5c). The high internal variability in rt203 explains why attempts to directly sequence the *psbA^ncr^* from this isolate ultimately failed. The presence of intragenomic variation may explain why the *psbA^ncr^* amplifies and/or sequences poorly for certain *ITS2* lineages (unpubl. data). While the *psbA^ncr^* of many *Symbiodinium* spp. exhibits relatively minor intragenomic variability and allows for unambiguous direct sequencing (see below), further investigations are needed to evaluate the range of genome contained variation across all the taxonomic ranks of this dinoflagellate group [20].

Some of the *psbA^ncr^* sequence variants recovered by cloning resulted from errors introduced during the PCR and cloning process (i.e., Taq polymerase error and/or mutations during cloning). The re-amplification, re-cloning, and sequencing of single amplicons produced variants that differed by a single base change (out of approx. 500 bp) from the original cloned sequence (Fig. 5c; JQ043720- JQ043745). Based on these examples, approximately 10 to 30% of cloned variants from original samples are probably artifacts of the method used to obtain these data (Fig. 5b), a finding which cautions against the over interpretation of small sequence differences present in data generated by bacterial cloning.

Intragenomic variation and methodological artifacts recovered by the PCR amplification and cloning of the *psbA^ncr^* appear minor when compared to the sequence divergence observed among haplotypes from different *Symbiodinium ITS2* lineages (Fig. 5a), and thus minimally interfere with phylogenetic signal provided by this marker. For both *psbA^ncr^* and *ITS2*, PCR/cloning retrieves rare intragenomic variants and generates sequence artifacts. However, *psbA^ncr^* exhibits much higher intergenomic variation between *Symbiodinium* types and species than does *ITS2*, and as a result, the confounding effects of intragenomic variation and methodological error are substantially reduced when analyzing *psbA^ncr^* data (see below).

**Figure 6:**
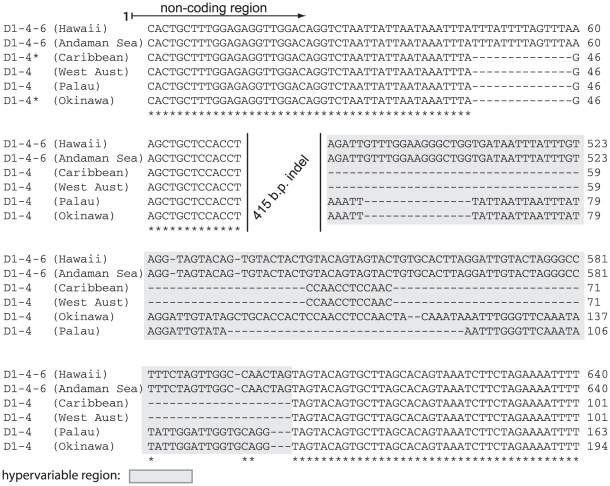
Alignment of partial *psbA* ^ncr^ sequences comparing *D1-4-6* with that of *Symbiodinium trenchi* (synonymous with *D1a* or *D1-4*) obtained from different hosts in geographically separate regions. Sequences marked by the asterisk are from isolates in culture.

Analysis of the *psbA* ^ncr^ was also applied to resolve contradictory interpretations pertaining to the identity of the Clade D *Symbiodinium* found in some colonies of *Montipora* spp. from Hawaii (see earlier discussion [22], [23]; Fig. 3). The chromatograms of *psbA* ^ncr^ directly sequenced from several samples were unambiguous (e.g., JQ043586), indicating that the *ITS2* variation observed in Figure 3 was representative of one entity (*sensu* [24]) and not a mixed community of Clade D symbionts as proposed by Stat et al. [23]. (A single Clade D multi-locus genotype was also detected in each sample based on the analysis of microsatellite markers [51], [52] [data not shown].) These sequences were nearly identical to the *psbA* ^ncr^ from samples of *Pocillopora* sp. collected in the Andaman Sea with the same *D1-4-6 ITS2*-DGGE profile (Fig. 6). These aligned poorly to partial *psbA* ^ncr^ sequences of *S. trenchi* (a.k.a. *D1a* or *D1-4*) found in samples collected from the western Pacific (*Lobophyllia hemprichii* from Palau) and eastern Indian Ocean (*Pocillopora damicornis* from Dampier, Western Australia; JQ043747), and cultured isolates from Okinawa, Japan and the Florida Keys, USA (A001, JQ003851, and 10.8b JQ043746, respectively) and therefore suggests that divergence time is much longer between these lineages than is indicated by the similarity in genomic composition of their dominant *ITS2* sequences.

### Phylogenetic utility of *psbA^ncr^*

**Figure 7:**
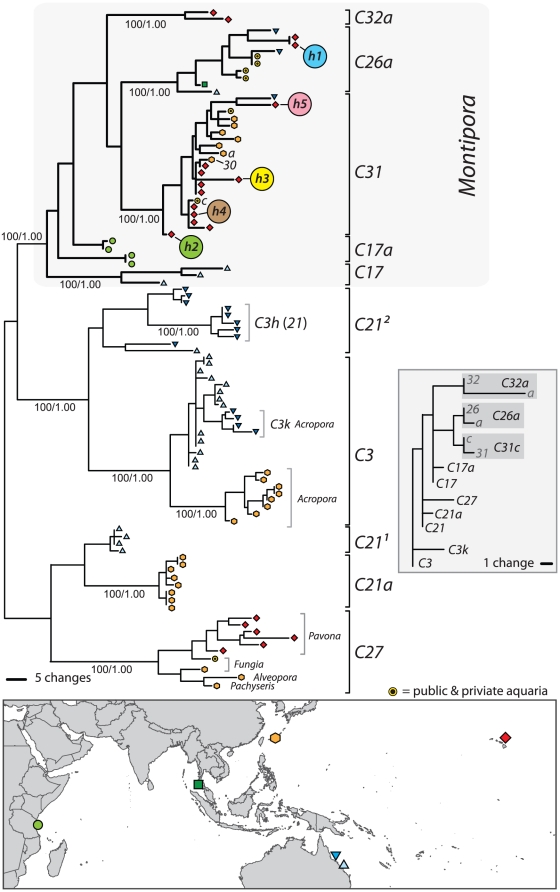
Phylogenetic reconstruction based on partial *psbA* ^ncr^ of common *Symbiodinium* ITS types characterized from locations throughout the Indo-Pacific (see map). Despite originating from geographically remote locations, *psbA* ^ncr^ haplotypes of particular ITS types group to form clades separated by considerable sequence divergence from other ITS types. A diverse monophyletic lineage consisting of different *ITS2* types associates exclusively with coral species in the genus *Montipora*. These are divergent from ‘ancestral’ host-generalist lineages *C3*, *C21*, and *C27*. The inset (shaded grey) is a corresponding *ITS2* phylogeny showing how, despite minimal sequence divergence, its topology reflects that of the *psbA* ^ncr^ tree.

The phylogeny of *psbA* ^ncr^ haplotypes in Figure 2a resolved three divergent lineages that corresponded with their respective *ITS2* membership. To assess whether the *psbA* ^ncr^ and *ITS2* (based on DGGE screening) data remain congruent over large geographic distances samples obtained from multiple regions throughout the Indo-Pacific were re-analyzed using both markers. These samples included representatives of types *C31* and *C26a* as well as *C3*, *C17*, and *C21*, lineages that allegedly exist within *M. capitata* from Hawaii [23]. Overall, the *psbA* ^ncr^ phylogeny was concordant with that of the *ITS2* phylogeny (Fig. 7 inset) with individual samples exhibiting reciprocal monophyly for each marker (JQ043587- JQ043676). *PsbA^ncr^* haplotypes of a particular *ITS2* type clustered together irrespective of geographic and host origin. For example, *Symbiodinium* designated as *C26a* from *Montipora* in the Indian Ocean, the Pacific Ocean, and the aquarium trade [42] formed a well-defined monophyletic cluster separated by large breaks in sequence similarity from other *ITS2* lineages. The one notable exception was that *psbA* ^ncr^ haplotypes designated “*C21*” formed two independent and divergent lineages not resolved by *ITS2* sequences. However these lineages are differentiated by *ITS1* sequences (data not shown) indicating that, with additional rDNA data, there is near complete correspondence between nuclear and plastid genomes [7], [53]. This concordance indicates sexual recombination in many cases appears limited to membership within an *ITS2* lineage and is grounds for defining biological species [54].

In comparison to the *psbA* ^ncr^, the *ITS2* is highly conserved and unchanging over broad geographic distances. *ITS2* lineages separated by small sequence differences are well differentiated by the numerous base substitutions and insertion-deletions in the *psbA* ^ncr^. These high amounts of sequence divergence revealed geographic partitioning not resolved by *ITS2* data. For example, the haplotypes of several *ITS2* lineages, including “*C3*” and “*C27*,” were sub-divided phylogenetically into populations originating from different biogeographic regions (Fig. 7). These data also indicate that small differences between *ITS* lineages mask extensive periods of elapsed time since their original divergence (millions of years in some cases [6]). *PsbA^ncr^* sequences from various *ITS* lineages comprising the “*C3* radiation” align poorly with sequences from lineages of the “*C1* radiation” (*sensu* [6], Fig. S1]) and suggests that a calibrated evolutionary clock for the rate of change in this plastid DNA would improve estimates of divergence times among the numerous “closely-related” lineages comprising *Symbiodinium* Clade C [6]. Although the *psbA^ncr^* is too divergent in certain cases (e.g., C1 vs. C2), when used in conjunction with more conserved DNA regions, the added genetic resolution offered by this marker will be extremely helpful in future investigations that require precise knowledge of *Symbiodinium* identity [17], [20].

### Symbiont homogeneity vs. heterogeneity in coral colonies

The proportion of colonies in a population found to harbor multiple *Symbiodinium* spp. is dependent on the species of host, reef habitat, and recent stress history [8], [40], [45], [55]–[58]. However, data from the *psbA* ^ncr^ appears consistent with observations that many coral colonies typically harbor a single dominant *Symbiodinium* genotype [8], [40], [51], [58]–[61]. While other species of symbiont are sometimes detectable at very low population densities, their ecological significance and temporal stability requires further evaluation [8], [43]. The argument that sequence variation in cloned rDNA is indicative of true biological diversity does not address the reality that extensive intragenomic variation and methodological artifacts account for most, if not all, sequence diversity in a sample [24]. If most colonies harbor highly-diverse *Symbiodinium* assemblages, then why did the cloning process employed here (Fig. 3), or by Stat et al. [23], fail to recover any of the *ITS2* and *psbA* ^ncr^ sequences that correspond to the many other *Symbiodinium* spp. (∼18) harbored by other cnidarians that co-occur with *Montipora capitata* in habitats around Kaneohe Bay [22]? Instead, Stat et al. [23] recovered most frequently the *ITS2* sequences diagnostic for *C31* and *D1-4-6*, and to a lesser extent sequences diagnostic of *Symbiodinium C21*. In addition, sequences for *C3* and C17, as well as 11 closely related novel variants, also were identified, that, with *C31* and *C21*, comprise a monophyletic lineage separate from all other Clade C *Symbiodinium* common among other corals in Hawaii [22]. If these sequences represent many different symbiont lineages, cloning and sequencing of the *psbA* ^ncr^ from *M. capitata* presumably would have detected multiple *psbA* ^ncr^ lineages corresponding to each of these different *Symbiodinium* spp. (e.g. *C21*) Because no such variation was detected here, the most parsimonious conclusion is that *Montipora* spp. coral colonies typically harbor one dominant genotype of one symbiont species (see discussion below). Future studies should use the *psbA* ^ncr^ to examine *Symbiodinium* diversity among the colonies of many species and from different regions to determine the generality of these findings. In this regard, rapidly evolving genetic markers with limited intragenomic variation, such as the *psbA* ^ncr^ and microsatellites, provide a third tier of differentiation (beyond Clades and ITS types) that resolve individual genotypes (or haplotypes) of *Symbiodinium* and can thus supply independent data on the relative homogeneity of a resident symbiont population.

The ribosomal array abounds with rare variants that are derived from the current numerically dominant copy, and yet may also contain once dominant ancestral sequences reduced in frequency by concerted evolution (Fig. 2a; [24]). The *psbA^ncr^* and *ITS2* phylogenies in Figure 7 offer a likely explanation for why *ITS2* sequences matching *C3*, *C17*, and *C21* (and minor variants of these) were obtained from cloning (Fig. 2a [23]). These three sequences are ancestral to the derived sequence “31” in the adaptive radiation of *Symbiodinium* Clade C associated with *Montipora* [6]. An ITS sequence is considered diagnostic of a particular *Symbiodinium* only if it is numerically dominant in the genome [7], [8], [24]. A rational explanation for the recovery of sequences that designate *C3*, *C21*, and *C17* during cloning is that these ancestral sequences persist in the genomes of *C31* individuals at low abundance and have not undergone complete replacement (i.e., lineage sorting) via concerted evolution (for example, see Fig. 6; [39]).

The sensitivity of bacterial cloning confounds analysis of rDNA because, aside from the sequence artifacts it introduces, low-copy variants are indiscriminately cloned and sequenced. That Stat et al. [23] reported significant sequence variation/variability at the colony level is not surprising and rather expected in the context of cloning's ability to recover rare variants. In theory, individuals in a species population should have essentially the same dominant *ITS* sequence (Fig. 7; [38]), but are likely to have considerable inter-individual variation involving low-abundance variants found throughout the ribosomal array of an individual's genome (a clonal lineage in the case of *Symbiodinium*). The findings of Stat et al. are more consistent with the reality that cloning rDNA and spacers mostly characterizes the variability of low-copy variants found among individual genotypes of *Symbiodinium C31* and *D1-4-6*.

### Conclusions and future considerations

**Figure 8:**
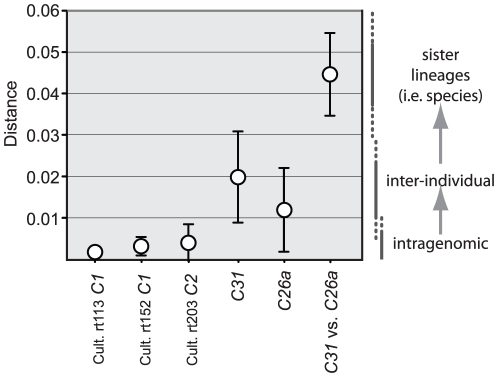
Average pairwise genetic distances compared among intragenomic, inter-individual, and inter-species sequence variation of the *psbA* ^ncr^ (error bars represent ± SD). Note the minimal overlap of nucleotide differences (Distance) between intragenomic (variation within a culture) and inter-individual variation (variation within an ITS2 lineage). This region is sometimes non-alignable among species lineages within a *Symbiodinium* Clade.

The *psbA^ncr^* is currently the fastest evolving genetic maker known for *Symbiodinium*, and provides independent evidence for assessing species diversity and ecology. The application of the *psbA^ncr^* in future investigations should significantly reduce uncertainty when interpreting the source of sequence variation. Unlike *ITS* rDNA, there appears to be considerably less overlap between intragenomic, inter-individual and inter-species variation (Fig. 8). Because of its fast evolutionary pace, the phylogenetic utility of the *psbA^ncr^* is limited to analyzing the relationships of only the most close-related species. Therefore, future studies should incorporate a hierarchical genetic approach to precisely examine the genetic diversity of *Symbiodinium* within and between colonies and across reef coral assemblages.

Conventional species descriptions for protists are presently impractical for the majority of *Symbiodinium*. This study demonstrates the efficacy of combining data from a multi-copy nuclear rDNA spacer and a rapidly evolving plastid marker to accurately discriminate species-level diversity in *Symbiodinium*. Indeed, the concordant patterns revealed by *ITS2* and *psbA^ncr^* (Fig. 7) show no recombination among sympatric lineages that, together with, well-supported monophyletic groupings with distinct ecological niches satisfy criteria according to the biological, phylogenetic, and ecological species concepts [14], [15]. We recommend that a combination of genetic and ecological data should supersede the conventional use of morphological features for describing species of *Symbiodinium*. The *psbA^ncr^* adds to a growing number of genetic tools that when used in combination provides the resolution necessary to thoroughly explore the global diversity, connectivity, and ecology of coral-*Symbiodinium* associations.

## Supporting Information

Figure S1 **Alignment of the complete** ***psbA^ncr^*** **sequence from the cultures rt113 (** ***Symbiodinium goreaui*** **), rt152 (** ***Symbiodinium goreaui*** **), and rt203 (** ***Symbiodinium*** **sp., type C2** ***sensu*** **LaJeunesse 2001) aligned using the online application of ClustalW2 (** http://www.ebi.ac.uk/Tools/msa/clustalw2/ **).** The cloned sequences chosen represent the numerically dominant sequence variant identified for each cultured strain.
(DOCX)

Table S1 ***Symbiodinium*** **samples, their ITS2 designations along with host species, depth of collection, and geographic origin.** GenBank accession numbers for *psbA^ncr^* sequences are provides and were used to reconstruct the phylogeny in Figure 7. Shallow, intermediate and deep refers to collection depths ranging from 1–5, 6–10, and >10 meters, respectively.
(DOC)
