# Supplementary material for: Improved Resolution of Reef-Coral Endosymbiont (Symbiodinium) Species Diversity, Ecology, and Evolution through psbA Non-Coding Region Genotyping
Source: PLoS One. 2011 Dec 28;6(12):e29013. doi: 10.1371/journal.pone.0029013 (PMC3247227; doi:10.1371/journal.pone.0029013)
Supplement: Figure S1 — Alignment of the complete psbAncr sequence from the cultures rt113 ( Symbiodinium goreaui ), rt152 ( Symbiodinium goreaui ), and rt203 ( Symbiodinium sp., type C2 sensu LaJeunesse 2001) aligned using the online application of ClustalW2 ( http://www.ebi.ac.uk/Tools/msa/clustalw2/ ). The cloned sequences chosen represent the numerically dominant sequence variant identified for each cultured strain. (DOCX) [file pone.0029013.s001.docx]

Supplemental Figure S1. Alignment of the complete *psbA^ncr^* nucleotide sequence from the cultures rt113 (*Symbiodinium goreaui*), rt152 (*Symbiodinium goreaui*), and rt203 (*Symbiodinium* sp., type C2 *sensu* LaJeunesse 2001). Sequences were aligned using the online application of ClustalW2 (<http://www.ebi.ac.uk/Tools/msa/clustalw2/>). The selected sequences represent the numerically dominant intragenomic variant from each cultured *Symbiodinium* strain.

Cult_113_clone4 TGTGGTTAGGG-TACCCTAATATTGGCCTGTTTTAGGTGCCTCTATGG-GTACTTCTATG 58

Cult_152_clone4 TGTGGTTAGGGGTACCCTAATATTGGCCTGTTTTAGGTGCCTCTATGG-GTACTTCTATG 59

Cult_203_clone5 ---TGTT-------CCTTTTTACAG--CTAATTTAC-TGCTTGCAAGCTGCAATTTT-TA 46

*** ** * ** * ** **** *** * * * * * ** * *

Cult_113_clone4 GGTACCAGCCTAATAATTTTGACATCAACCCTTTGATTTAGGGTACCCAGC-ATAGTACC 117

Cult_152_clone4 GGTACCAGCCTAATAATTTTGACATCAACCCTTTGATTTAGGGTACCCAGC-ATAGTACC 118

Cult_203_clone5 GCTGCTAGCCTGC--ATTTTTAGGCTGA-------AAATAGGCTTGCAAGCTGCAATTTG 97

* * * ***** ***** * * * **** * * *** * *

Cult_113_clone4 CA--TAAGGGGTGCCCTATTCCCCACACCCAAACAGGTACCCTAATTTAGACCAGAATTT 175

Cult_152_clone4 CA--TAAGGGGTGCCCTATTCCCCACACCCAAACAGGTACCCTAATTTAGACCAGAATTT 176

Cult_203_clone5 CAGCTAAAAATTAT--TATTTTTC---CTTAAAAA-------TAATTTTAGCTTGTGTTT 145

** *** * **** * * *** * ****** * * ***

Cult_113_clone4 AGGCCACATCATAAATTAGTGAGTTTTTTCACCCCGAAGGGATGGGTGCCCCTTGTGGGT 235

Cult_152_clone4 AGGCCACATCATAAATTAGTGAGTTTTTTCACCCCGAAGGGATGGGTGCCCCTTGTGGGT 236

Cult_203_clone5 TTGTT------TGAATTTTGGGGGTTT---AGCCCCAAAATATGGG--GCTTTAGC---- 190

* * **** * * *** * *** ** ***** * * *

Cult_113_clone4 ACCCATAT--GCCCGCAGGGCATTGAGGTCCACGAAGTGTGACCGTTAATTTTGGCCAAA 293

Cult_152_clone4 ACCCATAT--GCCCGCAGGGCATTGAGGTCCACGAAGTGTGACCGTTAATTTTGGCCAAA 294

Cult_203_clone5 -CCCATTCTGGCCCGAAGGAGCC---AGCCCCCGAAGGG-----GTGAA--------AAA 233

***** ***** *** * ** ***** * ** ** ***

Cult_113_clone4 AAA---GGGTGCCGCTAATTTTGGCCTA--AACTGCGACTATTTTGGCCAAAATTGCGAC 348

Cult_152_clone4 AAAA--GGGTGCCGCTAATTTTGGCCTA--AACTGCGACTATTTTGGCCAAAATTGCGAC 350

Cult_203_clone5 GAAATCGCAAGCCTGCGACT---GCCTGCTACCTGCGACTGCCT--GCTAC--CTGCGAC 286

** * *** * * **** * ******** * ** * ******

Cult_113_clone4 GTGTGTCCTCGCGCACACGCGCACACGTATTAAAAAGATCGCGACTTATCGTGAC--GTA 406

Cult_152_clone4 ATGTGTCCTCGCGCACACGCGCACACGTATTAAAAAGATCGCGACTTATCGTGAC--GTA 408

Cult_203_clone5 -TGCCTGCT-----ACCTGCGACTGCCTGCTAC-----CTGCGACTGCCTGCTACCTGCG 335

** * ** ** *** * * ** ****** * ** *

Cult_113_clone4 AATGGGTGCC-CCTGTGGGGCACCCATATGCACCACCCTTTGGGTGGTGCATTGGAGCCG 465

Cult_152_clone4 AATGGGTGCC-CCTGTGGGGCACCCATATGCACCACCCTTTGGGTGGTGCATTGGAGCCG 467

Cult_203_clone5 ACTGCCTGCTACCTGCAAGCCCGCAATTTTCACC------TGGGCCACAATTTTGGCCTC 389

* ** *** **** * * * ** * **** **** ** * *

Cult_113_clone4 GGAACG-AGCC-GAAGGCGAGTGGACGTGCGACCACGAAGAAAAGAAAAAGAAATCGCGA 523

Cult_152_clone4 GGAACG-AGCC-GAAGGCGAGTGGACGTGCGACCACGAAGAAAAGAAAAAGAAATCGCGA 525

Cult_203_clone5 GAAATGCAGCCTGCAGGCCCAAATAC---CAGCCGCAAATTTAGCA---AGAGATCGCGA 443

* ** * **** * **** ** * ** * ** * * *** *******

Cult_113_clone4 CCTATAATG-----GGTGCCCCTTTGGGGCACCCATATGCACACCCCGAAGGGGTGTGCA 578

Cult_152_clone4 CCTATAATG-----GGTGCCCCTTTGGGGCACCCATATGCACACCCCGAAGGGGTGTGCA 580

Cult_203_clone5 CTGATTAGGCCAAAATTGCCCCGAAGGGCAATTCACATGCGCGCGC-------ATGTGTG 496

* ** * * ****** *** * ** **** * * * ****

Cult_113_clone4 TTAATGGGCTGGGTGCCCTACCCAGCCCATATGGGCCCACGCTTCGCGGGGCCCATAACG 638

Cult_152_clone4 TTAATGGGCTGGGTGCCCTACCCAGCCCATATGGGCCCACGCTTCGCGGGGCCCATAACG 640

Cult_203_clone5 TAAAC-----ACATGCCCC-CGAAGGGGGCATGTGTCCA-GCCCCGAAGGGGCT------ 543

* ** ***** * ** *** * *** ** ** *** *

Cult_113_clone4 GCCCTTCGGGCCTTCAAAAAAAAAAAAGGTAATGA-TGGGTGCCAGTAACCGAGCAAAAT 697

Cult_152_clone4 GCCCTTCGGGCCTTCAAAAATAAAAAAG-TAATGA-TGGGTGCCAGTAACCGAGCAAAAT 698

Cult_203_clone5 -------GG---TTAAAAAAAAAAATATTTACCGAGTATGCGTC-GTG-CTGTGCGAGAA 591

** ** ***** **** * ** ** * * * * ** * * ** * *

Cult_113_clone4 AACCAAGCAAAT--CAACCTGCAGGCAATANTGGCCAAAACCTGTGCGAACCAAGTGTGA 755

Cult_152_clone4 AACCAAGCAAAT--CAACCTGCAGGCAATAATGGCCAAAACCTGTGCGAACCAAGTGTGA 756

Cult_203_clone5 TGCGACGCAAAGTGCGATTTGC--GCAATTTTGGCCTGATCAGTCGGGGTTTCAGTCAAA 649

* * ***** * * *** ***** ***** * * * * *** *

Cult_113_clone4 ATCTGCGGCTATGTCTCGGGGTGTGCATATGCC-TGCTGGCTGCCTGCATGCCACCTGCA 814

Cult_152_clone4 ATCTGCGGCTATGTCTCGGGGTGTGCATATGCC-TGCTGGCTGCCTGCA----------- 804

Cult_203_clone5 ATTTGCGGCTAGTTTTCGGGG-----ATTTGAGGTGAAAATTGCCTAAA----------- 693

** ******** * ****** ** ** ** ***** *

Cult_113_clone4 CATGTGCCCTCCACCCCTACCCCTCCTCCCAAC--AGAAGAATCTGGCCAAAATTATCAT 872

Cult_152_clone4 CATGTGCCCTCCACCCCTACCCCTCCTCCCAAC--AGAAGAATCTGGCCAAAATTATCAT 862

Cult_203_clone5 --------TTTCAGCTCAA-----------AATTAAGCAAAAACAGGTAAAAATTA--AT 732

* ** * * * ** ** * ** * ** ******* **

Cult_113_clone4 CACAATCTTTTGCAGGTGATTGATTACTACCAATGAAGAACACATCTT 920

Cult_152_clone4 CACAATCTTTTGCAGGTGATTGATTACTACCAATGAAGAACACATCTT 910

Cult_203_clone5 CACAATCTTTTGCAGGTGATTGATTACTACCAATGAAGAACACATCTT 780

************************************************
