# Supplementary material for: Improved Resolution of Reef-Coral Endosymbiont (Symbiodinium) Species Diversity, Ecology, and Evolution through psbA Non-Coding Region Genotyping
Source: PLoS One. 2011 Dec 28;6(12):e29013. doi: 10.1371/journal.pone.0029013 (PMC3247227; doi:10.1371/journal.pone.0029013)
Supplement: Table S1 — Symbiodinium samples, their ITS2 designations along with host species, depth of collection, and geographic origin. GenBank accession numbers for psbAncr sequences are provides and were used to reconstruct the phylogeny in Figure 7. Shallow, intermediate and deep refers to collection depths ranging from 1–5, 6–10, and >10 meters, respectively. (DOC) [file pone.0029013.s002.doc]

Table S1. *Symbiodinium* samples, their ITS2 designations along with host species, depth of collection, and geographic origin. GenBank accession numbers for *psbA^ncr^* sequences are provides and were used to reconstruct the phylogeny in Figure 7. Shallow, intermediate and deep refers to collection depths ranging from 1-5, 6-10, and > 10 meters, respectively.

| *Symbiodinium* ITS-DGGE type | *psbA^ncr^* GenBank accession number | Host Species | Region | Location | site | Collection Depth |
| --- | --- | --- | --- | --- | --- | --- |
|  |  |  |  |  |  |  |
| C32a | JQ043587 | *Montipora flabellata* | Central Pacific | Oahu | Kaneohe Bay, marker 9 | shallow |
| C32a | JQ043588 | *Montipora flabellata* | Central Pacific | Oahu | North shore | deep |
| C26a | JQ043590 | *Montipora incrassata* | Central Pacific | Oahu | Kaneohe Bay | deep |
| C26a | JQ043591 | *Montipora incrassata* | Central Pacific | Oahu | Kaneohe Bay | deep |
| C31 | JQ043608 | *Montipora pattula* | Central Pacific | Oahu | Kaneohe Bay | deep |
| C31 | JQ043612 | *Montipora capitata* | Central Pacific | Oahu | Kaneohe Bay | shallow |
| C31 | JQ043607 | *Montipora capitata* | Central Pacific | Oahu | Kaneohe Bay | shallow |
| C31 |  | *Montipora capitata* | Central Pacific | Oahu | North shore | deep |
| C31 | JQ043609 | *Montipora capitata* | Central Pacific | Oahu | North shore | deep |
| C31 | JQ043614 | *Montipora capitata* | Central Pacific | Oahu | Kaneohe Bay, marker 9 | shallow |
| C31 | JQ043615 | *Montipora capitata* | Central Pacific | Oahu | Kaneohe Bay, marker 9 | shallow |
| C31 | JQ043613 | *Montipora capitata* | Central Pacific | Oahu | Kaneohe Bay | shallow |
| C31 | JQ043610 | *Montipora capitata* | Central Pacific | Oahu | North shore | deep |
| C31 | JQ043611 | *Montipora capitata* | Central Pacific | Oahu | Kaneohe Bay | shallow |
| C31 | JQ043606 | *Montipora capitata* | Central Pacific | Oahu | Kaneohe Bay | intermediate |
| C26a | JQ043594 | *Montipora monasteriata* | West Pacific | GBR | Feather Reef North | shallow |
| C31 | JQ043598 | *Montipora turtlensis* | West Pacific | GBR | Feather Reef North | shallow |
| C26a | JQ043589 | *Montipora turtlensis* | West Pacific | GBR | Rib Reef North | deep |
| C21 | JQ043624 | *Leptoseris explanata* | West Pacific | GBR | Feather Reef South | deep |
| C21 | JQ043625 | *Pavona explanulata* | West Pacific | GBR | Feather Reef North | deep |
| C21 | JQ043626 | *Pachyseris speciosa* | West Pacific | GBR | Rib Reef North | deep |
| C3h | JQ043627 | *Leptoseris yabei* | West Pacific | GBR | Feather Reef North | deep |
| C3h | JQ043629 | *Fungia sp.* | West Pacific | GBR | Feather Reef North | deep |
| C3h | JQ043631 | *Pectinia paeonia* | West Pacific | GBR | Feather Reef North | deep |
| C3h | JQ043628 | *Turbinaria frondens* | West Pacific | GBR | Feather Reef North | deep |
| C3h | JQ043630 | *Podabacia crustacea* | West Pacific | GBR | Rib Reef North | deep |
| C21 | JQ043632 | *Acropora latistella* | West Pacific | GBR | Curacao Island | intermediate |
| C3K | JQ043645 | *Acropora humilis* | West Pacific | GBR | Feather Reef North | shallow |
| C3K | JQ043647 | *Acropora florida* | West Pacific | GBR | Feather Reef North | shallow |
| C3K | JQ043646 | *Acropora nasuta* | West Pacific | GBR | Feather Reef North | shallow |
| C3K | JQ043648 | *Acropora nobilis* | West Pacific | GBR | Rib Reef North | shallow |
| C27 | JQ043667 | *Pavona varians* | Central Pacific | Oahu | North shore | deep |
| C27 | JQ043672 | *Pavona duerdeni* | Central Pacific | Oahu | North shore | deep |
| C27 | JQ043673 | *Pavona duerdeni* | Central Pacific | Oahu | North shore | deep |
| C27 | JQ043671 | *Pavona varians* | Central Pacific | Oahu | Kaneohe Bay, marker 9 | shallow |
| C27 | JQ043674 | *Pavona varians* | Central Pacific | Oahu | Kaneohe Bay, marker 9 | shallow |
| C27 | JQ043668 | *Pavona duerdeni* | Central Pacific | Oahu | Kaneohe Bay | deep |
| C17 | JQ043618 | *Montipora monasteriata* | West Pacific | GBR | Heron Island | deep |
| C17 | JQ043620 | *Montipora monasteriata* | West Pacific | GBR | Heron Island | deep |
| C17 | JQ043619 | *Montipora aequituberuculata* | West Pacific | GBR | Heron Island | deep |
| C26a | JQ043593 | *Montipora stellata* | West Pacific | GBR | Heron Island | shallow |
| C3 | JQ043635 | *Platygra daedalea* | West Pacific | GBR | Heron Island | shallow |
| C3i | JQ043649 | *Acropora tenuis* | West Pacific | GBR | Heron Island | shallow |
| C3 | JQ043634 | *Acropora clathroata* | West Pacific | GBR | Heron Island | shallow |
| C3 | JQ043639 | *Acropora cerealis* | West Pacific | GBR | Heron Island | shallow |
| C3 | JQ043640 | *Acropora gemmifera* | West Pacific | GBR | Heron Island | deep |
| C21 | JQ043633 | *Acrhelia horresans* | West Pacific | GBR | Heron Island | shallow |
| C3 | JQ043643 | *Acropora palifera* | West Pacific | GBR | Heron Island | shallow |
| C3 |  | *Acropora polystoma* | West Pacific | GBR | Heron Island | shallow |
| C3 | JQ043644 | *Acropora tenuis* | West Pacific | GBR | Heron Island | shallow |
| C3 | JQ043641 | *Acropora microclados* | West Pacific | GBR | Heron Island | shallow |
| C3 | JQ043642 | *Acropora nasuta* | West Pacific | GBR | Heron Island | shallow |
| C3 | JQ043636 | *Favia stellegera* | West Pacific | GBR | Heron Island | shallow |
| C3 | JQ043637 | *Favia abdita* | West Pacific | GBR | Heron Island | shallow |
| C3 | JQ043638 | *Goniastrea aspera* | West Pacific | GBR | Heron Island | shallow |
| C21 | JQ043658 | *Echinophyllia echinata* | West Pacific | GBR | Heron Island | shallow |
| C21 | JQ043659 | *Echinophyllia echinata* | West Pacific | GBR | Heron Island | deep |
| C21 | JQ043660 | *Echinopora lamellosa* | West Pacific | GBR | Heron Island | deep |
| C21 | JQ043661 | *Merulina ampliata* | West Pacific | GBR | Heron Island | deep |
| C21a | JQ043664 | *Lobophyllia robusta* | West Pacific | Ryukyus Isls. | Zamami Island | shallow |
| C21a | JQ043663 | *Echinophyllia sp.* | West Pacific | Ryukyus Isls. | Zamami Island | shallow |
| C3 | JQ043650 | *Acropora copiosa* | West Pacific | Ryukyus Isls. | Zamami Island | shallow |
| C3i | JQ043654 | *Acropora valida* | West Pacific | Ryukyus Isls. | Zamami Island | shallow |
| C3i | JQ043651 | *Acropora donei* | West Pacific | Ryukyus Isls. | Zamami Island | shallow |
| C3 | JQ043652 | *Acropora secale* | West Pacific | Ryukyus Isls. | Zamami Island | shallow |
| C3 | JQ043655 | *Acropora nobilis* | West Pacific | Ryukyus Isls. | Zamami Island | shallow |
| C3 | JQ043656 | *Acropora cerealis* | West Pacific | Ryukyus Isls. | Zamami Island | shallow |
| C3 | JQ043653 | *Acropora valida* | West Pacific | Ryukyus Isls. | Zamami Island | shallow |
| C3 | JQ043657 | *Acropora cerealis* | West Pacific | Ryukyus Isls. | Zamami Island | shallow |
| C30 | JQ043605 | *Montipora efflorescens* | West Pacific | Ryukyus Isls. | Zamami Island | shallow |
| C31 | JQ043616 | *Montipora sp.* | West Pacific | Ryukyus Isls. | Zamami Island | shallow |
| C31 | JQ043603 | *Montipora sp.* | West Pacific | Ryukyus Isls. | Zamami Island | shallow |
| C31 | JQ043600 | *Montipora capitata* | West Pacific | Ryukyus Isls. | Zamami Island | shallow |
| C31 | JQ043604 | *Montipora venosa* | West Pacific | Ryukyus Isls. | Zamami Island | intermediate |
| C31 | JQ043599 | *Montipora sp.* | West Pacific | Ryukyus Isls. | Zamami Island | intermediate |
| C31a | JQ043617 | *Montipora sp.* | West Pacific | Ryukyus Isls. | Zamami Island | intermediate |
| C21a | JQ043662 | *Pectinia sp.* | West Pacific | Ryukyus Isls. | Zamami Island | intermediate |
| C21a |  | *Pectinia alcicornis* | West Pacific | Ryukyus Isls. | Zamami Island | intermediate |
| C21a | JQ043665 | *Symphillia radians* | West Pacific | Ryukyus Isls. | Zamami Island | intermediate |
| C21a | JQ043666 | *Symphillia radians* | West Pacific | Ryukyus Isls. | Zamami Island | intermediate |
| C21a |  | *Favites halicora* | West Pacific | Ryukyus Isls. | Zamami Island | intermediate |
| C21a |  | *Favites halicora* | West Pacific | Ryukyus Isls. | Zamami Island | intermediate |
| C27 | JQ043669 | *Alveopora sp.* | West Pacific | Ryukyus Isls. | Zamami Island | intermediate |
| C27 | JQ043670 | *Pachyseris speciosa* | West Pacific | Ryukyus Isls. | Zamami Island | intermediate |
| C27 | JQ043676 | *Fungia sp.* | West Pacific | Ryukyus Isls. | Zamami Island | intermediate |
| C26a | JQ043595 | *Montipora sp.* | Indo-Pacific | Aquarium | unknown | unknown |
| C26a | JQ043596 | *Montipora aequituberculata* | Indo-Pacific | Aquarium | unknown | unknown |
| C26a | JQ043592 | *Montipora capricornis* | Indo-Pacific | Aquarium | unknown | unknown |
| C26a |  | *Montipora capricornis* | Indo-Pacific | Aquarium | unknown | unknown |
| C31 | JQ043602 | *Montipora sp.* | Indo-Pacific | Aquarium | unknown | unknown |
| C31c | JQ043601 | *Montipora capitata* | Indo-Pacific | Aquarium | unknown | unknown |
| C27 | JQ043675 | *Fungia sp.* | Indo-Pacific | Aquarium | unknown | unknown |
| C17a | JQ043621 | *Montipora sp.* | Western Indian Ocean | Zanzibar | Banda Kuu | Intermediate |
| C17a | JQ043622 | *Montipora undata* | Western Indian Ocean | Zanzibar | Banda Kuu | Intermediate |
| C17a | JQ043623 | *Montipora sp.* | Western Indian Ocean | Tanzania | Tanga | Intermediate |
| C17a |  | *Montipora sp.* | Western Indian Ocean | Tanzania | Tanga | Intermediate |
| C26a | JQ043597 | *Montipora sp.* | Northeastern Indian Ocean | Andaman Sea | Similan Islands | Intermediate |
